# Genetic Diversity of *Rhodiola quadrifida* (Crassulaceae) in Altai High-Mountain Populations of Kazakhstan

**DOI:** 10.3390/genes16121449

**Published:** 2025-12-03

**Authors:** Oxana Khapilina, Ainur Turzhanova, Moldir Zhumagul, Saule Magzumova, Olesya Raiser, Damelya Tagimanova, Serik Kubentayev, Vladislav Shevtsov

**Affiliations:** 1 National Center for Biotechnology, Astana 010000, Kazakhstan; turzhanova-ainur@mail.ru (A.T.); magzumovas@list.ru (S.M.); 2008olesya@mail.ru (O.R.); tagds@mail.ru (D.T.); xatabadich@gmail.com (V.S.); 2 Astana Botanical Garden, Astana 010000, Kazakhstan; mzhakypzhan@mail.ru (M.Z.); kubserik@mail.ru (S.K.); 3 Department of Graduate School of Natural Sciences, Astana International University, Astana 010000, Kazakhstan

**Keywords:** *Rhodiola quadrifida* (Pall.) Fisch. & C.A. Mey., DNA molecular marker, genetic diversity, iPBS amplification

## Abstract

**Background:** *Rhodiola quadrifida* (Pall.) Fisch. & C.A. Mey. (Crassulaceae) is a rare medicinal species in the Kazakh Altai, yet information on its population structure and genetic diversity remains limited. This study presents findings from an investigation of natural *R. quadrifida* populations. **Methods:** The morphometric characteristics, population age structure, and genetic diversity of the plants were analysed using PCR-based genome profiling. Genetic diversity within *R. quadrifida* populations was assessed using PCR primers for binding sites complementary to a specific region at the 3′ terminus of a particular tRNA. **Results:** The populations exhibited variations in morphological traits, age structures, and reproductive strategies. The IVA-1, IVA-2 and KOK populations represent the most mature stages, characterized by a dominance of vegetative reproduction and a disturbed age structure, with a predominance of senile and virgin individuals. In contrast, the LIN-1 and LIN-2 population is characterized by a balanced age structure, encompassing all ontogenetic groups, and a mixed reproductive system that includes both sexual and vegetative propagation. Genetic diversity, as measured by Shannon’s information index, ranged from 0.194 to 0.247, indicating low genetic diversity in *R. quadrifida*. Analysis of molecular variance (AMOVA) revealed significantly greater variation within populations (62%) than among populations (38%). Genetic diversity was higher in the LIN-1 and LIN-2 populations, which employs a mixed reproductive system (clonal and seeds), than in populations dominated by vegetative reproduction. Both LIN populations, characterized by a mixed reproductive system, exhibited higher genetic diversity than the KOK, IVA-1 and IVA-2 populations, where vegetative reproduction predominated. **Conclusions:** These results underscore the necessity for priority conservation measures, including monitoring population size and age structure in populations with low levels of seed reproduction and disturbed age structure. Additional measures include supporting *in situ* and *ex situ* conservation, such as clonal collection, seed banks, and tissue cultures, as well as restricting the harvesting of medicinal raw materials.

## 1. Introduction

The diverse genus *Rhodiola* L. (Crassulaceae family) represents a taxonomically complex and ecologically significant group, comprising approximately 70 species. The centres of species diversity for this genus are predominantly located in the mountainous and arid regions of Asia, with 55 species, including 16 endemic species found in China and several species distributed across Northeast Asia and Central Asia, including the Tien Shan and Altai regions. Numerous species of the *Rhodiola* genus exhibit valuable medicinal properties and are extensively utilized in both conventional and academic medicine, thereby heightening interest in their study. However, this increased interest poses a threat to natural populations [1,2,3,4].

Recent research has focused on other *Rhodiola* species prevalent in Kazakhstan, such as *Rhodiola quadrifida* (Pall.) Fisch. & C.A. Mey. (Crassulaceae) is a rare plant with adaptive properties. This species inhabits alpine zones, channeries, moss-and-lichen tundra, rocky hill slopes, and debris near glaciers, resulting in low seed productivity and survival rates for young *R. quadrifida* plants. The species is found at elevations ranging from 1800 to 4000 m above sea level, with summer temperatures between 2 and 15 °C. *R. quadrifida* is distributed in the high mountain areas of Russia (Altai, Sayan mountains), as well as in the mountainous regions of China and Mongolia. Kazakhstan is confined to the ridges of Kazakhstani Altai and Tarbagatai.

This plant is of particular interest because of its unique and understudied chemical composition, which includes flavonoids, coumarins, salidroside, and thyrozol [5,6]. Phenylethanoids, such as mongrhoside, which has a psychostimulant effect, and rhodiocyanoside, a cyanogenic glycoside, have been identified in the roots of *R. quadrifida* [7]. The underground parts of *R. quadrifida* are sources of gossypetin glycosides (rhodiolgyne and rhodioflavonoside), tricetin hydroxynitrile glycosides, and essential oils. The chemical composition of *R. quadrifida* essential oils includes acids such was hexadecanoic acid (45%), 9,12-octadecanoic acid (33%), and 9-hexadecenoic acid (3%). Information on the chemical composition of the aboveground portions of the species is limited to the presence of quercetin and kaempferol [8]. *R. quadrifida* extracts exhibit significant pharmacological activity and are employed in traditional Chinese medicine as haemostatic, antitussive, and tonic agents for treating gynecological pathologies and urinary diseases [9,10,11]. Additionally, *R. quadrifida* extracts have been found to inhibit angiogenic processes in primary tumour tissues, significantly reducing the likelihood of cancer metastasis [9]. The potential for producing salidroside and rosavin in callus and suspension cultures in vitro, as well as in hairy root cultures, has also been explored [12].

A critical area of focus in the study of rare and endangered plant species is the comprehensive assessment of genetic diversity and the status of natural populations, which includes intrapopulation polymorphisms and the rate of genetic differentiation between populations. When combined with morphological analysis and the study of population biological characteristics and age structure, this approach allows for thorough characterization of a species and informs the selection of optimal strategies for the preservation and sustainable use of the gene pool. Molecular genetic markers are instrumental in evaluating the status of rare and endangered species under anthropogenic and climatic conditions [13]. These markers facilitate the identification of the genetic structure of populations, determination of genetic diversity levels both within and between populations, and development of efficient, scientifically grounded resource protection strategies [14]. Currently, representatives of the genus *Rhodiola* are being studied using PCR-based molecular marker systems. Notably, the use of Random Amplified Polymorphic DNA (RAPD)-related PCR methods, such as inter simple sequence repeat (ISSR) genome profiling, is well established for effectively evaluating genetic diversity and population structure [15,16,17]. Furthermore, systematic and phylogenetic studies have frequently employed markers based on nuclear ribosomal DNA sequences, particularly internal transcribed spacers (ITS), as well as plastid DNA regions such as trnL-F and psbA-trnH, which have demonstrated their informativeness in resolving phylogenetic relationships between species [17].

To investigate the genetic diversity of rare species, it is advantageous to employ markers derived from interspersed repeat sequences such as mobile genetic elements or other repetitive elements [18,19,20,21]. These repeated sequences are prevalent in genomes and exhibit high levels of representation and polymorphism, which is particularly crucial when alternative markers are unavailable for analysis of a specific species [22,23,24,25,26]. The most comprehensive approach is inter-primer binding site (iPBS) PCR genome profiling, which utilizes conserved regions of the primer binding site (PBS) [27]. PBS is a conserved region on the LTR-retrotransposon RNA, complementary to the 3′ end of the tRNA that initiates reverse transcription during the retrotransposon replication cycle [22]. PBS sequences are both conserved and universal, enabling the use of a single primer for amplification of interspersed repeats and other interspersed repeat sequences [27]. This method uncovers molecular genetic polymorphisms resulting from retrotransposons and generates reproducible PCR fingerprint profiles during electrophoresis on agarose gels [28].

This iPBS profiling technology was effectively employed to examine the Kazakhstani population of *Rhodiola* sp. It has been demonstrated that a high degree of differentiation is associated with both resistance to adverse environmental conditions and evolutionary divergence, as well as characteristics of the reproductive system. In the case of the *R. linearifolia* population, a correlation was established between the genetic polymorphism of retrotransposons and metabolome profile [29,30]. Studies on the genetic diversity of *R. quadrifida* were conducted on a single population and addressed the issues of interspecies interaction of *Rhodiola* sp. within the context of reproductive organization characteristics, specifically monoecy and dioecy [31]. However, the relationship between the propagation system (seed and/or vegetative) and genetic polymorphism in mountainous populations of *R. quadrifida* remains unexplored. ISSR-marker studies of *R. alsia* populations on the Tibetan Plateau indicate that a significant portion of genetic variability arises from inter-population polymorphism and vegetative propagation, whereas periodic sexual reproduction may occur to maintain high levels of intrapopulation diversity. A similar reduction in genetic diversity was observed in populations of other *Rhodiola* species, which consist of reproductively mature plants resulting from outbreeding or clonal propagation [32,33]. It is posited that the greater the contribution of sexual reproduction, the higher the intrapopulation diversity and adaptive potential, the lower the intrapopulation polymorphism, and the greater the distinction between populations [34,35]. Investigating this interconnection is critical for medicinal plants, which represent a vulnerable category of biological resources. Intensive, irrational, and inadequately regulated collection of raw plant materials exerts a destabilizing impact on the natural populations of *R. quadrifida*. The genetic diversity and reproductive strategies of *R. quadrifida* have not been extensively investigated. There is limited understanding of the mechanisms by which populations engage in both vegetative and sexual reproduction, and how these strategies correlate with genetic variability as revealed through iPBS profiling. This study aims to elucidate the relationship between reproductive strategies and genetic diversity in *R. quadrifida*, which is crucial for evaluating the current status of the species and for formulating conservation strategies in response to anthropogenic pressures and changing habitats.

## 2. Materials and Methods

### 2.1. Plant Material

This study concentrated on *R. quadrifida* specimens collected from Eastern Kazakhstan, specifically within the Southern and Western Altai ranges. All plant specimens referenced in this article, including *R. quadrifida*, were identified by the study’s author, S.A. Kubentayev, and Moldir Zhumagul. The herbarium voucher specimens have been deposited in the Herbarium of the Astana Botanical Garden under the numbers NUR 007586–007594. The species *R. quadrifida* is not subject to protection within the territory of Kazakhstan. The research was conducted on public lands without protected status; consequently, no special permits for plant collection or field studies were necessary.

**Figure 1:**
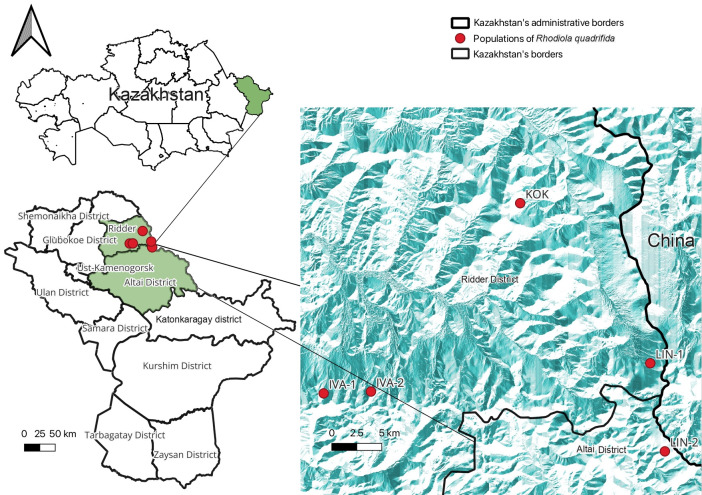
Location of populations of *R. quadrifida* in Kazakhstani Altai.

The species populations were characterized by isolated microphytocenoses ranging from 150 to 1500 m^2^. The coordinates and absolute elevation of the population locations where the plant material was collected were determined using a global positioning system (GPS) navigator (Table 1, Figure 1). The density of the aboveground cover was assessed following the methodology [36]. The habitat characteristics of *R. quadrifida* populations are detailed in Table 1.

### 2.2. Morphometric Characteristic Analysis of R. quadrifida Plants

Ten adult generative specimens were selected for each population. The following morphological parameters were measured for each plant: plant height (cm), bush diameter (cm), number of sprouts per bush, inflorescence diameter (cm), and number of flowers per inflorescence (items). Each sample was assigned a unique identification number, collection location, coordinates (latitude and longitude), and type. The structure of each population was analyzed using the method outlined by Rabotny [37]. The methodologies of Uranov were employed to investigate the life cycle [38].

### 2.3. Genetic Analysis

DNA samples from 10 mature plants per population were used for genetic analysis. Genomic DNA was isolated from homogenized samples using a high-salt gel electroelution trap or acidic CTAB solution (2% CTAB, 2 M NaCl, 10 mM Na3EDTA, and 50 mM HEPES, pH 5.3) [37,39,40]. The extracted DNA was subsequently dissolved in 1× TE buffer (1 mM EDTA, 10 mM Tris-HCl, pH 8.0), the DNA concentration was determined spectrophotometrically using a Nanodrop instrument (Thermo Fisher Scientific, Waltham, MA, USA), and qualitative assessment was performed via gel electrophoresis. For the analysis of genetic diversity within *R. quadrifida* populations, primers developed by Kalendar [27] were employed.

The PCR mixture, with a total volume of 25 µL, contained 10 ng of DNA, 1xPhire buffer, 1 µM primer, 200 mM dNTP, and 1U Phire Hot Start II DNA Polymerase (Thermo Fisher Scientific). The amplification protocol included initial denaturation at 98 °C for 1 min, followed by 32 cycles of amplification at high annealing temperatures of 55–60 °C for 30 s and 72 °C for 60 s, and a final elongation at 72 °C for 2 min. Amplification was conducted using 11 primers in a SimpliAmp Thermal Cycler (Thermo Fisher Scientific, Waltham, MA, USA). The primer sequences used are listed in Table 2. The amplification results were confirmed using 1.5% agarose gel supplemented with ethidium bromide in 1x TAE buffer. The sizes of the iPBS fragments were determined by comparison with the DNA Ladder Mix 100–10,000 bp (Thermo Fisher Scientific GeneRuler). The lengths of the resulting iPBS fragments were visualized using an iBright 1500 Imaging System (Invitrogen, Carlsbad, CA, USA).

### 2.4. Statistical Analysis

To identify interpopulation differences based on morphological traits, a one-way analysis of variance (ANOVA) was performed using Rstudio (version 2023.3.0.386). In instances where significant differences were observed (*p* < 0.05), a post hoc Tukey test (Tukey HSD) was conducted. Mean values are presented as mean ± standard error (SE). STRUCTURE version 2.3.4 software was utilized to ascertain the hierarchical organization of the genetic structure of the 11 populations, with the admixture model selected as the ancestry model. This Bayesian-based software estimates the number of genetic clusters (K) and evaluates the admixture levels among them. Analysis was performed for K values ranging from 1 to 10. Each run included a burn-in period of 100,000 iterations, followed by 500,000 Markov Chain Monte Carlo (MCMC) iterations [42]. The dataset comprises 69 individuals genotyped at 47 loci. The number of genetic groups (K) was determined using the Evanno’s method [43]. A graphical visualization of the population structure was generated using the CLUMPAK online resource [44]. Diversity characteristics of the genetic data were calculated using GenAlEx v.6.5 software [45]. The number of alleles (Na), effective number of alleles (Ne), Shannon information index (I), expected heterozygosity (He), unbiased expected heterozygosity (uHe), and polymorphic locus percentage (P) were determined for each locus.

## 3. Results

### 3.1. Evaluation of R. quadrifida Plant Morphometric Characteristics

**Figure 2:**
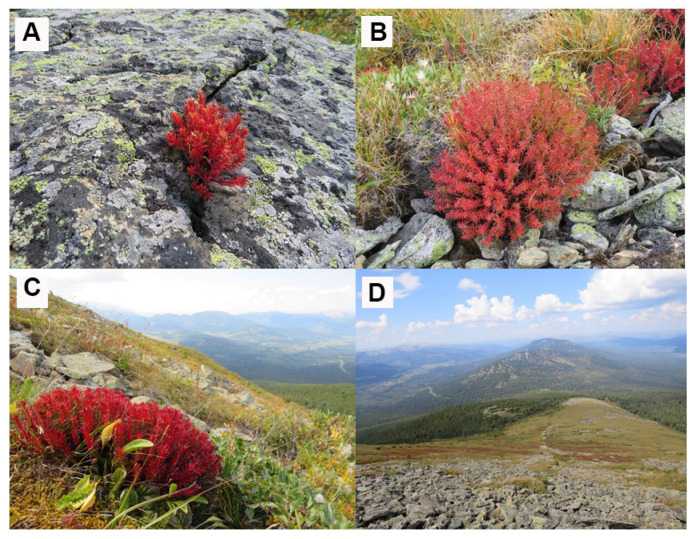
*R. quadrifida* plants in the Kazakhstani Altai. (**A**) plants of *R. quadrifida* in IVA-1 population; (**B**) plants of *R. quadrifida* in LIN-1 population; (**C**) plants of *R. quadrifida* in LIN-2 population; (**D**) overview of a *R. quadrifida* LIN-1 population in situ (Photo by Premina N.).

The habitats of the species are situated in alpine and mountain-tundra zones at elevations ranging from 1800 to 2400 m above sea level. *R. quadrifida* predominantly thrives on residual hills, rocks, micro- and macro-fragmental rockslides, as well as in moss-and-lichen, dryad, and stony sedge tundra (Figure 2). This species is frequently found in biocenoses along with *Carex capituliformis*, *C. capillaris*, *C. rupestris*, *Dryas oxyodonta*, *Bergenia crassifolia*, *Aster alpinus*, *Patrinia sibirica*, *Saxifraga sibirica*, *Astragalus alpinus*, *Festuca kryloviana*, *F. borissii*, *Viola biflora*, *Schulzia crinita*, *Hedysarum austrosibiricum*, *Thermopsis alpina*, and *Gentiana grandiflora*. It forms pioneer micropopulations on rockslides and rocks, with vegetative reproduction prevailing across all the examined biocenoses. The in vivo ground-germination rate of this species was approximately 75%. Owing to harsh habitat conditions, seed propagation is limited, with nearly 85% of seedlings perishing after the first overwintering.

The IVA-1 population is located on the northwestern slope of the Ivanovsky Ridge within Dryas-sedge associations on rocky and crushed-stony slopes at the summit of an ancient moraine, where winter snow cover is nearly absent, leading to frost damage to renewal buds. The projective cover of *R.a quadrifida* accounted for no more than 3–5%. The ground cover was well-developed, reaching 100% coverage. The plants were in a suppressed state, with sporadic flowering. The bushes were predominantly aged and easily fragmented into separate clones. The wintering buds were small and the root system was underdeveloped. The absence of a standing grass crop exacerbates substrate erosion and exposes renewed buds, resulting in winterkills. The IVA population is ageing and is sustained through ineffective vegetative propagation with a disturbed age composition.

Population IVA-2 is situated on the rocky massif of the Ivanovsky Ridge in the upper reaches of the Bolshaya Poperechka River on a steep north-facing slope. The species inhabits partially overgrown ledges, rock fissures, and spaces between fragments of ancient moraines. The substrate was composed of clay gravel dominated by flat gravel pieces. The ground cover is well-developed, reaching up to 100%, and is primarily formed by mosses up to 5 cm thick and lichens. The plant community involving *R. quadrifida* is well-developed, with a total projective cover of 10–15%. The *R. quadrifida* population consists predominantly of adult individuals with low seed regeneration. Reproduction is mainly vegetative; as the central part of the tuft ages, it disintegrates into small clones that are transported to other sites by snowmelt or rockfall.

The KOK population is situated on the southwestern slope of the Koksinsky Ridge, near the Saugin-Kamen rock, as part of a mixed-herb pioneer phytocenosis on a partially overgrown mountain moraine. The total projective coverage was 35%. The ground cover is well developed, comprising mosses and lichens, with coverage reaching up to 90%. *R. quadrifida* grows in polydominant groups along rock fissures and cavities between parent rock debris. The population is young and has emerged because of seed drift from external sources. Fruit formation is limited, with only up to 12% of flowers developing into follicles. Most inflorescences desiccate, likely because of frost damage to the buds at an early developmental stage. This population of *R. quadrifida* is of the normal type and is capable of self-renewal through both seed and vegetative propagation.

The LIN-1 population was located on the northeastern slope of the Lineysky Ridge. The total projective cover is 5–7%. The ground cover was well developed, with a coverage of up to 95%. *Rhodiola* grew diffusely throughout the group area and its periphery. Seed-bearing is weak and occurs only in plants under the canopy of stone blocks. The growth conditions are extreme; the plants are low-growing, not exceeding 11 cm in height, but possess large buds covered by dense leathery scales that protect developing organs from adverse factors. Seed production is weak and occurs only in boulder shelters. Despite the presence of numerous juveniles and generative plants, their survival rates are low. The population was fully structured and normal.

The LIN-2 population is located on the southwestern slope of the Lineysky Ridge, near the Latunikha River. The ground cover was poorly developed, with coverage not exceeding 30%, consisting mainly of mosses and lichens. The vegetation is weakly expressed, occurring in patches or strips, with a total projected cover of 5–7%. *Rhodiola* grows diffusely on the exposed boulders on the leeward side. The population is fully structured, of the normal type; sprouts are numerous but have a low survival rate. The LIN-2 population exhibited the most balanced age composition; almost all age groups were represented, with juvenile sprouts prevailing. Consequently, the population can self-renew through both seed and vegetative propagation.

**Figure 3:**
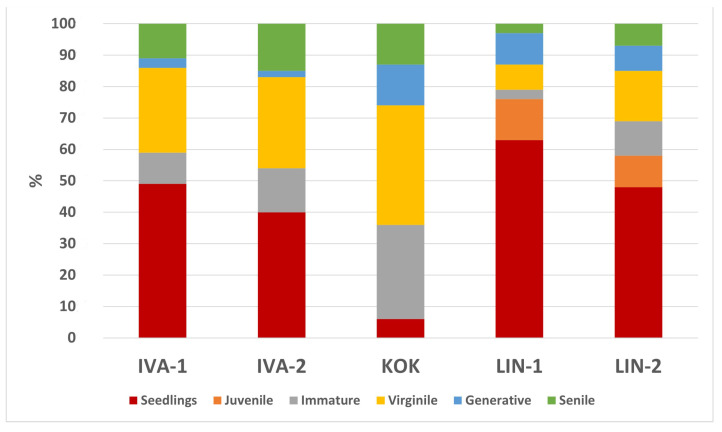
Age structure of *R. quadrifida* populations.

The morphological and quantitative characteristics of the aboveground parts of *R. quadrifida* revealed differences in the main criteria used to assess the external structure of the plants, as well as in the age structure of the population and the predominant modes of reproduction (Figure 3).

Analysis of the age structure of *R. quadrifida* populations indicated that only the LIN-1 and LIN-2 populations exhibited a structure encompassing all age groups, with a substantial proportion of seedlings, ranging from 48% in LIN-2 to 63% in the LIN-1 population. Immature plants were present across all populations, with the highest proportion observed in KOK. The proportion of virgin plants was relatively consistent across all populations, although it peaked in IVA-1, IVA-2, and KOK, with values of 27%, 29%, and 38%, respectively. The proportion of generative individuals demonstrated considerable variability among the populations; they were rare or nearly absent in IVA-1 and IVA-2 but constituted a significant share in the LIN-1, LIN-2, and KOK populations at 10%, 8%, and 13%, respectively. Morphometric comparisons of the populations (Figure S1) revealed distinct differences in vegetative and reproductive traits. Comparative analysis of the morphological characteristics among the three populations showed statistically significant differences in several parameters (Table 3).

One-way analysis of variance revealed significant differences in most morphological traits among the studied populations. Plant height exhibited significant variation among populations (*p* < 0.05). The tallest plants were observed in the KOK and IVA-2 populations, measuring 9.58 cm and 9.59 cm, respectively, while the shortest height was recorded in the IVA-2 population at 6.23 cm. Bush diameter also varied among the populations, with the maximum value characteristic of the IVA-2 population at 25.4 cm, whereas the minimum bush diameter was recorded for the KOK population at 9.58 cm. The number of shoots per bush demonstrated pronounced variability (*p* < 0.001); in the IVA-2 and KOK populations, the values were high at 45.1 and 43.6, respectively, whereas in the LIN-1 and LIN-2 populations, they were nearly half as much, at 24.4 and 26.5, respectively. The inflorescence diameter differed significantly between populations, ranging from 1.69 to 1.97 cm (*p* < 0.05). In contrast, the number of flowers in the inflorescence varied significantly between populations, with the KOK population exhibiting the highest mean value at 6.86, whereas the IVA-2 population had a noticeably lower number of flowers (3.19). Additionally, analysis of variance indicated that certain morphological traits of *R. quadrifida* were significantly influenced by environmental variables (Table 3).

The studied factors had the most significant effect on the number of shoots and the number of flowers (*p* < 0.001); no effect was detected on plant height or inflorescence diameter (*p* > 0.05). The greatest variability of morphometric traits in *R. quadrifida* plants was found depending on slope exposure (Table 4).

**Figure 4:**
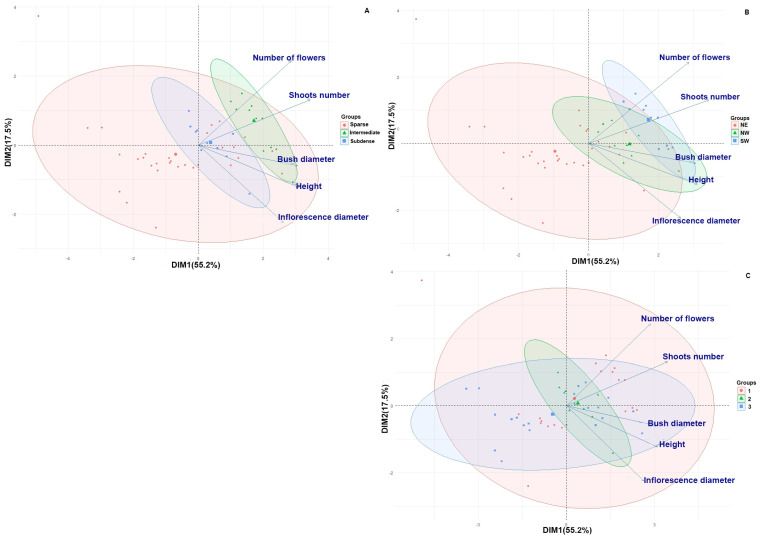
Principal Component Analysis of morphological variation in *R. quadrifida*. Points represent individual plants, coloured by ecological grouping factor; ellipses indicate 95% confidence ranges. Panels: (**A**) Coverage, (**B**) Slope, (**C**) Vegetation group.

Principal Component Analysis (PCA) of morphological traits revealed consistent ordination patterns across population, slope, and vegetation groups, as PCA was conducted on the complete trait matrix. PC1 accounted for 55.2% of the total variance, while PC2 accounted for 17.5%, representing 72.7% of the total variability (Figure 4).

Principal Component 1 (PC1) was correlated with the overall plant size, encompassing height, bush diameter, and number of shoots. In contrast, Principal Component 2 (PC2) represented variation in reproductive traits, primarily the number of flowers and inflorescence diameter. The findings indicated varying degrees of influence from factors such as total projective cover, slope orientation, and soil cover intensity on the morphometric characteristics of the plants. Notably, the highest values of the biometric characteristics of *Rhodiola* plants were observed on slopes oriented to the southwest, characterized by well-developed soil cover and high total projected cover. Conversely, *R. quadrifida* plants from sparse communities or north-facing slopes exhibited smaller bush sizes and fewer shoots.

### 3.2. PBS-Profiling of R. quadrifida

For amplification, 11 primers were employed, which demonstrated reproducible and distinct bands that were subsequently included in further analysis. Electrophoretic separation of PCR products yielded amplicons ranging from 100 to 5000 base pairs (bp) in size. All primers were highly informative, with the most informative markers being 2221 (0.900), 2230 (0.920), 2241 (0.920), and 2395 (0.930) (Table S1). All primers were deemed reliable for genetic polymorphism analysis, particularly markers with Polymorphic Information Content (PIC) greater than 0.90. Unique profiles consisting of clear and assessable bands were generated for each sample, with their distribution profiles contingent on the specific population and primers used. The profiles obtained were analyzed based on the presence or absence of bands across the three populations of *R. quadrifida* (Figures S2–S12). Electrophoretic separation of the PCR products facilitated the acquisition of amplicons ranging from 100 to 4500 bp. The genetic diversity indices for the *Rhodiola* populations analyzed are presented in Table 5. The genetic variability of *R. quadrifida* populations (Table 5) revealed significant differences in genetic diversity parameters. LIN-1 and LIN-2 populations exhibited the highest genetic diversity, with the highest number of alleles (Na) recorded at 1.427 and 1.512, respectively. Indicators such as the number of effective alleles (Ne), Shannon’s information index, and expected heterozygosity were also the highest in these populations. These populations also exhibited the highest percentages of polymorphic loci at 67% and 72%, respectively. In contrast, the KOK and IVA-2 populations demonstrated the lowest levels of genetic variability, with Na values of 1.041 and 1.020, respectively, and expected heterozygosity (He) values of 0.128 and 0.124, respectively. The IVA-1 population displayed intermediate values for most of the parameters. Overall, the mean percentage of polymorphic loci across all populations was 58.13%, indicating a moderate level of genetic diversity within the studied *R. quadrifida* populations. Analysis of Molecular Variance (AMOVA) revealed that 43% of the total genetic variability was attributable to differences between populations, whereas 57% was distributed within populations (Table 6).

**Figure 5:**
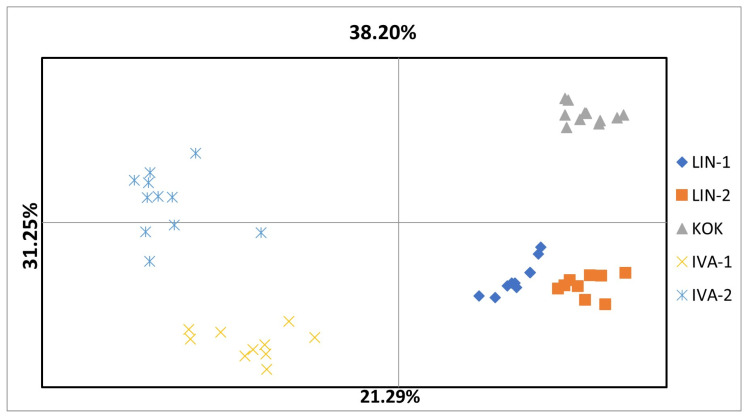
Principal Coordinates Analysis (PCoA) of Wild *R. quadrifida* populations.

The estimated variance components were 20.261 among the populations and 27.393 within the populations, contributing to a total variance of 47.654. Overall genetic differentiation was substantial, as evidenced by a PhiPT value of 0.425 (*p* < 0.01). Pairwise Nei’s genetic distances among *R. quadrifida* populations suggested moderate to high genetic similarity across the studied groups (Table 7). The smallest genetic distance was observed between LIN-1 and LIN-2 (0.921), whereas IVA-1 and IVA-2 also exhibited close genetic proximity (0.913). Conversely, the greatest genetic differentiation was identified between KOK and LIN-1 (0.747) and between KOK and IVA-2 (0.866), indicating reduced genetic exchange or increased isolation between these populations. The genetic structure of *R. quadrifida* was assessed using Principal Coordinate Analysis (PCoA) and cluster analysis based on iPBS profiles. Analysis of the principal components revealed that the first three axes collectively accounted for 90.74% of the total variation (Figure 5).

**Figure 6:**
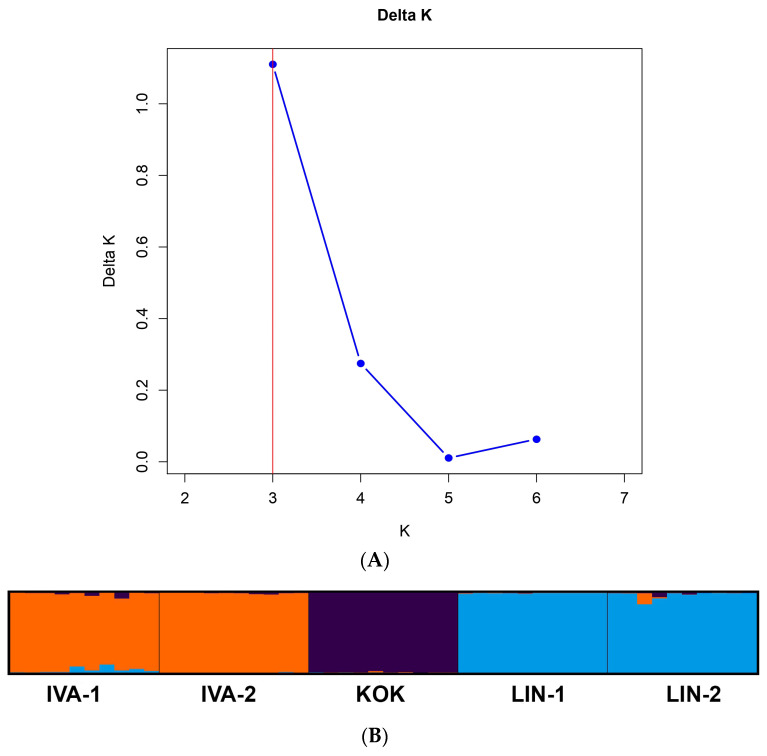
Results of STRUCTURE analysis of five *R. quadrifida* populations. (**A**) Delta K values determined by Structure Harvester for various numbers of populations assumed (K) in STRUCTURE analysis. (**B**) Each colour represents a different genetic cluster, and the number of groups (K) was set to 4 based on the method described by Evanno et al. The length of the coloured segment indicates the estimated membership proportion of individuals in the designed group.

The first two coordinates accounted for 38% and 31% of the total genetic variation, respectively, cumulatively explaining 69% of the variation. Populations LIN-1 and LIN-2 formed closely related clusters along the positive axis of PCoA1, indicating a high degree of genetic similarity. In contrast, IVA-1 and IVA-2 were positioned on the opposite side of PCoA1, reflecting substantial genetic divergence from LIN populations. The KOK population is distinctly separated. Bayesian clustering analysis was conducted to assess the structure of the *R. quadrifida* populations. The optimal number of genetic clusters was determined as previously described by Evanno et al. The highest ΔK value was observed for K = 3 (Figure 6). The assignment probabilities of individuals to these clusters revealed clear genetic differentiation among the populations. Populations IVA-1 and IVA-2 were predominantly assigned to the first cluster, KOK formed a separate and genetically distinct group corresponding to the second cluster, and populations LIN-1 and LIN-2 were mainly associated with the third cluster. Only a small degree of admixture was detected in the populations.

## 4. Discussion

*R. quadrifida* is a valuable adaptogenic species that is prevalent in the mountainous and cold regions of Asia. Its composition is under active investigation, with evidence supporting its angiogenic, antioxidant, and strengthening effects. However, for its comprehensive medicinal application, further studies on natural populations, which serve as primary sources of medicinal raw materials, are necessary. The genetic diversity of natural populations is crucial for the adaptability and preservation of valuable and endangered plants, underscoring the need for such studies. To examine the interspecific and population diversity of *Rhodiola* sp., various molecular markers have been employed, including RAPD, SSR, ISSR, AFLP, and iPBS [17,22,25,27,28]. Nonetheless, the *R. quadrifida* population remains insufficiently studied and is one of the most under-studied species within the Kazakhstani flora. This species is associated with mountainous habitats such as mountainous tundras and subalpine meadows, which are particularly vulnerable to climatic changes [46,47]. An additional threat to the preservation of the species is intensive harvesting for use in folk medicine, potentially leading to the loss of local genotypes and a reduction in the adaptive potential of the population. The results obtained indicated significant differences among natural populations of *R. quadrifida* concerning both morphological variability and genetic diversity, as revealed through iPBS profiling. Morphometric comparisons among populations (Figure S1) demonstrated distinct differences in both vegetative and reproductive traits.

Factors such as slope orientation, soil cover intensity, and overall canopy cover had a significant influence on the biometric characteristics of *R. quadrifida*, as shown by Principal Component Analysis (PCA), *R. quadrifida* plants growing on southwest- and northwest-facing slopes were characterized by greater bush height, a higher number of shoots, and more flowers. This may be related to differences in the daily pattern of insolation and the radiation balance of the underlying surface on slopes with different orientations, since areas of maximum soil heating systematically shift following the apparent movement of the sun [48].

In contrast, northeast-facing slopes receive reduced radiation during summer, and in winter, the irradiation of these slopes approaches zero [49]. Adaptation to specific local conditions determines the variability of morphological parameters, as confirmed by the results of one-way ANOVA (*p* < 0.001).

Analysis of the age structure of *R. quadrifida* populations showed that the LIN-1 and LIN-2 populations had a higher number of young plants, indicating the presence of both sexual (seed) and vegetative reproduction. In contrast, the KOK, IVA-1, and IVA-2 populations were dominated by adult plants, with a significantly lower number of shoots and young individuals, which may be due to the predominance of vegetative reproduction, wherein the central part of the herbaceous turf gradually decays with age and fragments into smaller clones. These clones disperse to other areas via snow avalanches or landslides. In such habitats, seed reproduction is challenging; seeds often lack sufficient time to produce viable sprouts, and young seedlings are susceptible to perishing because of late spring and summer frosts, as well as snowfall. Sprouts predominantly perish in spring. An additional limiting factor is the requirement for prolonged seed stratification, approximately 10–11 months at specific temperatures [50]. The probable absence of seedlings may also result from the extended germination dynamics common to high mountainous species. For instance, in *R. algida*, which grows in the foothills of Altai, seeds germinate over 2–3 subsequent growing seasons, serving as an adaptation strategy to severe high mountainous conditions [47]. In extremely unfavourable habitats, vegetative propagation is an effective and preferred life cycle strategy for sustaining population, where populations accumulate only competitive genotypes adapted to stressful conditions [51,52,53].

The maximum values of seedlings and plants in the juvenile and generative stages in LIN-1 and LIN-2 populations indicate the presence of a mixed propagation system, encompassing both seed and vegetative methods. The employment of diverse propagation strategies mitigates the effects of stress factors, ensures genetic variability, reduces population susceptibility, and facilitates expansion into new environmental niches [52]. It is possible that the KOK population originated from seed dispersal, most likely from the LIN-1 population. Relatively favourable conditions, such as a southwest-facing slope and dense above-ground cover, create optimal microrelief conditions for the development of *R. quadrifida* plants. Despite the predominance of vegetative propagation in the IVA and KOK populations, it cannot be concluded that all the plants are genetically uniform. This was corroborated by the results of iPBS profiling, which generated a unique and unmatched amplification profile for each population. The iPBS markers not only revealed the diversity of individuals within *R. quadrifida* populations, but also confirmed the polymorphism of retrotransposon allele variations, which can potentially influence genes related to adaptation responses. In the context of population variability, new cis-regulatory sequences may emerge in *R. quadrifida* plants, which are potentially significant for evolutionary processes and enable new forms to adapt to extreme habitat conditions [54,55]. Natural barriers, such as mountain ranges and woodlands, significantly reduce gene flow between populations, and the dominance of vegetative propagation also limits genetic diversity and our findings are consistent with those of previous studies that employed various marker systems for other *Rhodiola* sp. [27,34,36]. iPBS profiling analysis revealed a low genetic diversity in the IVA population, which predominantly consists of ageing generative plants, is likely attributable to the absence of seed regeneration. Reproductively mature plants resulting from outbreeding or clonal propagation in natural populations of *R. dumulosa*, *R. granulata*, and *R. sachalinensis* also exhibit low diversity [32,46,49]. Partitioning of genetic divergence using AMOVA revealed that a substantial portion of genetic variability, specifically 57%, was attributed to intrapopulation variability. The data obtained were consistent with previous results for other plant species taxa, including *Rhodiola*, where intrapopulation variability was significantly higher than interpopulation variability [3,29,31,33].

The observed variations in genetic polymorphism levels, identified using iPBS markers, underscore the necessity for further investigation and monitoring of *R. quadrifida* populations. Such efforts will not only enhance our understanding of the species’ adaptive strategies but also establish a scientific foundation for its conservation and sustainable utilization as a source of medicinal raw materials. Our results highlight the critical importance of preserving and judicious utilization of *R. quadrifida* requires a regular monitoring of population size and structure, particularly for the KOK, IVA-1, and IVA-2 populations, which exhibit signs of ageing and reduced seed renewal rates. At the same time, the pronounced ecological and biological conservatism of *R. quadrifida* prevents its effective conservation under ex situ conditions. A long-term attempt to cultivate this species in the Altai Botanical Gardens have been unsuccessful, underscoring the continued reliance on natural populations as the primary source of medicinal raw materials [54,55]. In this context, preserving the biodiversity of *R. quadrifida* necessitates a comprehensive strategy that includes both in situ (such as protecting natural habitats, regulating plant harvesting, and reintroduction efforts within the species’ natural range) and ex situ measures (such as establishing seed banks and in vitro collections). Future research should aim to integrate genetic, metabolomic, and ecological data to achieve a more comprehensive understanding of the mechanisms driving the adaptation of species to extreme alpine environments and evaluate the effects of climate change on population dynamics. This approach provides a scientific basis for developing programmes for the long-term preservation of *R. quadrifida* and its sustainable use in medicine and pharmaceuticals.

## 5. Conclusions

The findings of our study indicate substantial morphological and genetic differentiation among natural populations of *R. quadrifida*, which reflect the impact of local environmental conditions and diverse reproductive strategies. These observed differences underscore the uneven adaptive potential of populations and the susceptibility of those lacking seed-based regeneration. The application of iPBS markers has proven effective for genetic analyses of *R. quadrifida* populations with varying reproductive strategies. This retrotransposon-based method not only uncovered genetic variability among the populations studied but also provided valuable insights that can enhance the broader understanding of the adaptive potential of *R. quadrifida*. The predominance of intrapopulation variability and the low level of genetic diversity are critical factors contributing to the species’ vulnerability, highlighting the necessity for ongoing population monitoring in the development of conservation strategies. Future research should aim to integrate genetic, ecological, and physiological approaches to elucidate the mechanisms underlying the adaptation of *R. quadrifida* to extreme high-mountain conditions.

## Figures and Tables

**Table 1:** Ecological and phytocenotic conditions of *R. quadrifida* populations in the Kazakhstani Altai.

Population	Phytocenosis	Gathering Place	Coordinates		Height Limit, m Above Level m	Projective Cover (%)	Slope	Ground Cover (%)
			Latitude	Longitude				
IVA-1	*Dryas oxydontha*, *Carex capillaris*, *Carex rupestris*, *Viola biflora*, *Thermopsis alpina*, *Silene graminifolia*, *Koenigia alpina Minuartia verna*, *Patrinia sibirica*.	Ivanovsky ridge	50°18′41.1	83°47′19.0	1900–2400	45,780 (Sparse)	NW	100 (Dense)
IVA-2	*Anemonastrum crinitum*, *Bistorta elliptica*, *Dracocephalum grandiflorum*, *Pachypleurum alpinum*, *Bergenia crassifolia*, *Gentiana algida*, *G. grandiflora*, *Oxytropis alpina*, *Hedysarum austrosibiricum*, *Festuca borissii*, *F. kryloviana*, *Thermopsis alpina*, *Dryas oxyodonta*, *Patrinia sibirica*, *Huperzia selago*, *Woodsia heterophylla*, *Valeriana capitata*, *Dracocephalum imberbe*, *Betula fruticosa*.	Ivanovsky ridge, upper reaches of the Bolshaya Poperechka river	50°18′47.1	83°51′18.1	2000–2300	42,278 (Sparse)	NE	100 (Dense)
KOK	*Hierochloe odorata*, *Silene graminifolia*, *Hedysarum austrosibiricum*, *Dryas oxyodonta*, *Trisetum spicatum*, *Koeleria ledebourii*, *Gentiana algida*, *Bistorta vivipara*, *Thermopsis alpina*, *Pedicularis oederi*, *Chulzia crinita*, *Patrinia sibirica*, *Pachypleurum alpinum*, *Allium pumilum*, *Eremogone formosa*, *Lloydia serotina*.	Koksinsky ridge	50°28′53.5	84°03′49.8	1950–2000	35 (Intermediate)	SW	90 (Dense)
LIN-1	*Aster alpinus*, *Poa attenuata*, *Bergenia crassifolia*, *Bupleurum longiinvolucratum*, *Epilobium lactiflorum*, *Patrinia sibirica*, *Allium lineare*, *Ligularia glauca*, *Aconitum anthoroideum*, *Sedum ewersii*, *Bistorta elliptica*.	Lineisky ridge	50°20′18.4	84°14′46.1	1830–2200	50 (Subdense)	NE	95 (Dense)
LIN-2	*Bupleurum longiinvolucratum*, *Gentiana algida*, *G. grandiflora*, *Crepis chrysantha*, *Schulzia crinita Pedicularis amoena*, *P. oederi*, *Huperzia selago*, *Dracocephalum grandiflorumpeжe Silene graminifolia*, *Dracocephalum imberbe*, *Pachypleurum alpinum*, *Bergenia crassifolia*.	Lineisky ridge, Latunikha River	0°15′33.8	84°15′59.7	2139	5–7 (Sparse)	SW	30 (Sparse)

**Table 2:** PBS primer sequences used for PBS profiling of *R. quadrifida*.

Primer ID	Sequence	Tm °C	GC%	LC%
2220	ACCTGGCTCATGATGCCA	55	55.6	86
2221	ACCTAGCTCACGATGCCA	55	55.6	92
2222	ACTTGGATGCCGATACCA	53	50.0	90
2228	CATTGGCTCTTGATACCA	55	44.4	90
2229	CGACCTGTTCTGATACCA	53	50.0	90
2230	TCTAGGCGTCTGATACCA	53	50.0	94
2232	AGAGAGGCTCGGATACCA	55	55.6	86
2240	AACCTGGCTCAGATGCCA	55	55.6	87
2241	ACCTAGCTCATCATGCCA	53	50.0	89
2300	CACCGGGCTCTGATACCA	57	61.1	90
2395	TCCCCAGCGGAGTCGCCA	62	72.2	82

Note: Tm °C—annealing temperature, Linguistic Complexity (LC) [41].

**Table 3:** One-way ANOVA with Tukey HSD post hoc test results for morphological traits of three populations of *R. quadrifida*.

Population	Plant Height	Bush Diameter	Number of Shoots per Bush	Inflorescence Diameter	Number of Flowers in an Inflorescence
IVA-1	8.28 ± 1.36	17.6 ± 2.56	45.7 ± 3.39	1.97 ± 0.24	4.74 ± 0.62
IVA-2	6.23 ± 0.25	12.7 ± 0.50	36.4 ± 2.91	1.71 ± 0.15	3.19 ± 0.27
KOK	9.58 ± 0.89	15.1 ± 1.03	45.1 ± 3.58	1.97 ± 0.36	6.86 ± 0.33
LIN-1	8.67 ± 1.16	17.3 ± 1.43	24.4 ± 3.05	1.69 ± 0.28	4.87 ± 0.46
LIN-2	9.59 ± 0.31	12.4 ± 0.69	26.5 ± 1.58	1.80 ± 0.13	4.54 ± 0.43
*p* value	<0.001	<0.001	<0.001	<0.05	<0.001

Note: Values are presented as mean ± SD.

**Table 4:** Summary of one-way ANOVA and Tukey HSD results testing effects of environmental factors on morphological traits of *R. quadrifida*.

Trait	Projection Coverage F (*p*); Tukey sig.	Slope F (*p*); Tukey Sig.	Ground Vegetation F (*p*); Tukey Sig.
Height	2.10 (*p* > 0.05);	0.83 (*p* > 0.05);	1.42 (*p* > 0.05);
Bush diameter	1.22 (*p* > 0.05);	**4.09 (*****p***** < 0.05)**; NW > NE	**4.16 (*****p***** < 0.05)**; Dense > Sparse
Shoot number	**13.43 (*****p***** < 0.001)**; Int > Sparse; Int > Subdense	**64.67 (*****p***** < 0.001)**; NW > NE; SW > NE	**20.80 (*****p***** < 0.001)**; Dense > Sparse
Inflorescence diameter	1.87 (*p* > 0.05);	1.91 (*p* > 0.05);	0.56 (*p* > 0.05);
Flowers number	**9.78 (*****p***** < 0.001)**; Int > Sparse; Int > Subdense	**8.48 (*****p***** < 0.001)**; SW > NE; SW > NW	0.10 (*p* > 0.05);

Note: Significant ANOVA results (*p* < 0.05) are shown in bold. Height and inflorescence diameter showed no significant responses to any of the tested environmental factors.

**Table 5:** Genetic variation in the analyzed *R. quadrifida* populations.

Pop	Na	Ne	I	He	uHe	*p* (%)
IVA-1	1.264	1.239	0.247	0.154	0.162	60.98
IVA-2	1.020	1.198	0.194	0.124	0.130	45.12
KOK	1.041	1.204	0.200	0.128	0.134	45.53
LIN-1	1.427	1.349	0.324	0.212	0.223	67.07
LIN-2	1.512	1.342	0.320	0.207	0.218	71.95
Mean	1.253	1.266	0.257	0.165	0.173	58.13

Note: Na: number of alleles per locus; Ne: effective number of alleles; I: Shannon’s information index; He: expected heterozygosity; uHe: unbiased expected Heterozygosity; PB: number (%) of polymorphic loci.

**Table 6:** Analysis of molecular variance (AMOVA) based on PBS-profiling data.

Source	df	SS	MS	Est. Var.	%	PhiPT
Among Pops	4	920.000	230.000	20.261	43%	0.425
Within Pops	45	1232.700	27.393	27.393	57%	
Total	49	2152.700		47.654	100%	

**Table 7:** Pairwise Nei genetic distances for *R. quadrifida* populations.

IVA-1	IVA-2	KOK	LIN-1	LIN-2	
1.000					IVA-1
0.913	1.000				IVA-2
0.880	0.866	1.000			KOK
0.821	0.773	0.747	1.000		LIN-1
0.828	0.783	0.779	0.921	1.000	LIN-2

## Data Availability

The original contributions presented in this study are included in the article/Supplementary Materials. Further inquiries can be directed to the corresponding author.

## Supplementary Materials

The following supporting information can be downloaded at https://www.mdpi.com/article/10.3390/genes16121449/s1. Figure S1: Morphological profile of *Rhodiola quadrifida* in Eastern Kazakhstan populations; Table S1: Polymorphism and PIC Statistics for iPBS primers. Figures S2–S12: inter Primer Binding site (iPBS) profiling of individual DNA samples from *R. quadrifida* populations.

## Abbreviations

PBS: primer binding site; LTR: long terminal repeat.
