# Supplementary material for: Genetic Diversity of Rhodiola quadrifida (Crassulaceae) in Altai High-Mountain Populations of Kazakhstan
Source: Genes (Basel). 2025 Dec 3;16(12):1449. doi: 10.3390/genes16121449 (PMC12732715; doi:10.3390/genes16121449)
Supplement: Supplementary file 1 [file genes-16-01449-s001.zip › genes-4020437-supplementary.pdf]

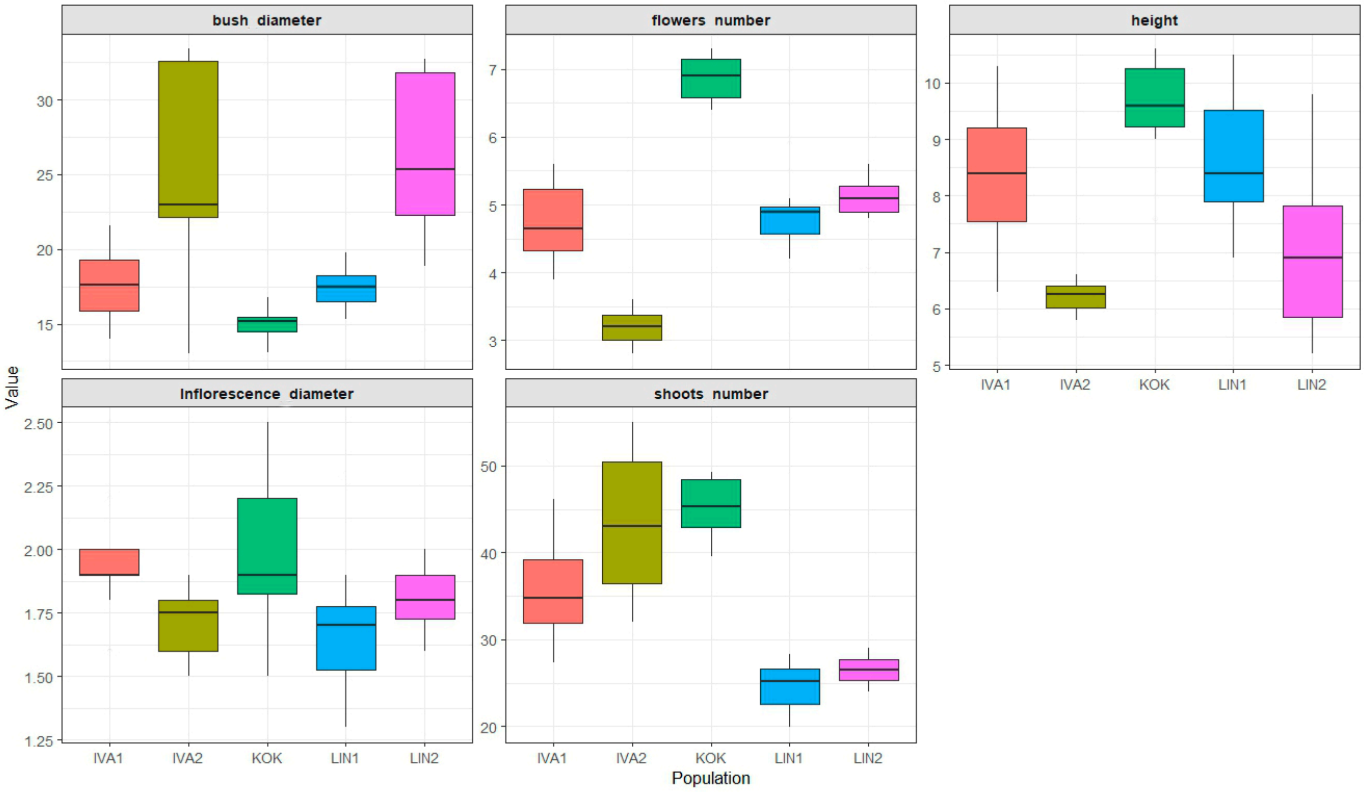

Figure S1 – Morphological profile of *Rhodiola quadrifida* in Eastern Kazakhstan populations

Table S1 – Polymorphism and PIC Statistics for iPBS primers

№	Primer ID	Total bands	Polymorphic bands (%)	PIC
1	2220	379	54,29	0.84
2	2221	267	95,00	0.90
3	2222	585	84.00	0.91
4	2228	260	36,00	0.65
5	2229	300	43,00	0.77
6	2230	251	82.76	0.92
7	2232	141	46,40	0.82
8	2240	445	54,29	0.89
9	2241	446	81,76	0.91
10	2300	280	65.00	0.88
11	2395	384	88,13	0.93

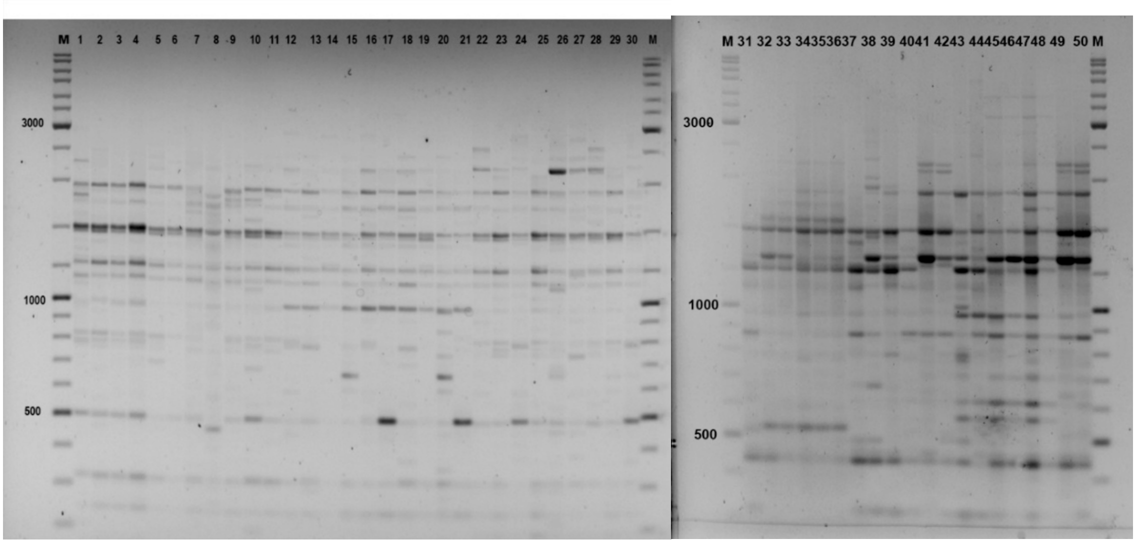

Figure S2 – inter Primer Binding Site (iPBS) profiling of individual DNA samples from *R. quadrifida* populations using primer 2220. M - Thermo Scientific GeneRuler DNA Ladder Mix (100–10,000 bp). Samples: 1-10 IVA-1 population, 11-20 IVA-2 population, 21-30 КОК population, 31-40 LIN-1 population, 41-50 LIN-2 population.

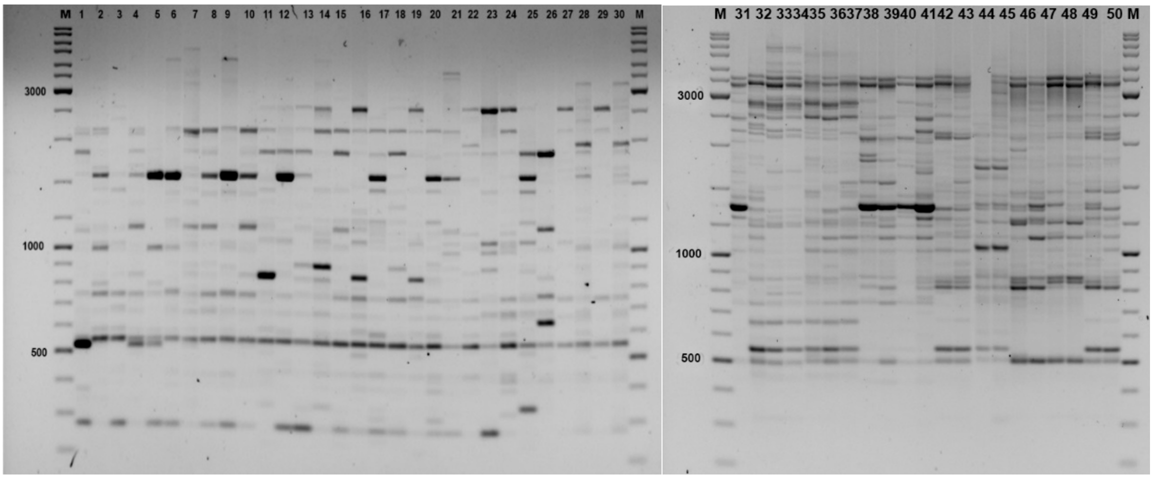

Figure S3 – inter Primer Binding Site (iPBS) profiling of individual DNA samples from *R. quadrifida* populations using primer 2221. M - Thermo Scientific GeneRuler DNA Ladder Mix (100–10,000 bp). Samples: 1-10 IVA-1 population, 11-20 IVA-2 population, 21-30 КОК population, 31-40 LIN-1 population, 41-50 LIN-2 population.

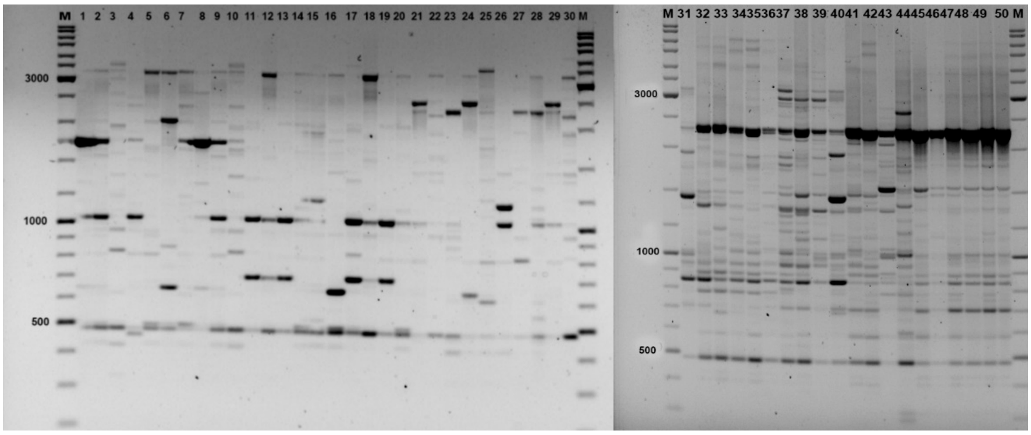

Figure S4– inter Primer Binding Site (iPBS) profiling of individual DNA samples from *R. quadrifida* populations using primer 2222. M - Thermo Scientific GeneRuler DNA Ladder Mix (100–10,000 bp). Samples: 1-10 IVA-1 population, 11-20 IVA-2 population, 21-30 КОК population, 31-40 LIN-1 population, 41-50 LIN-2 population.

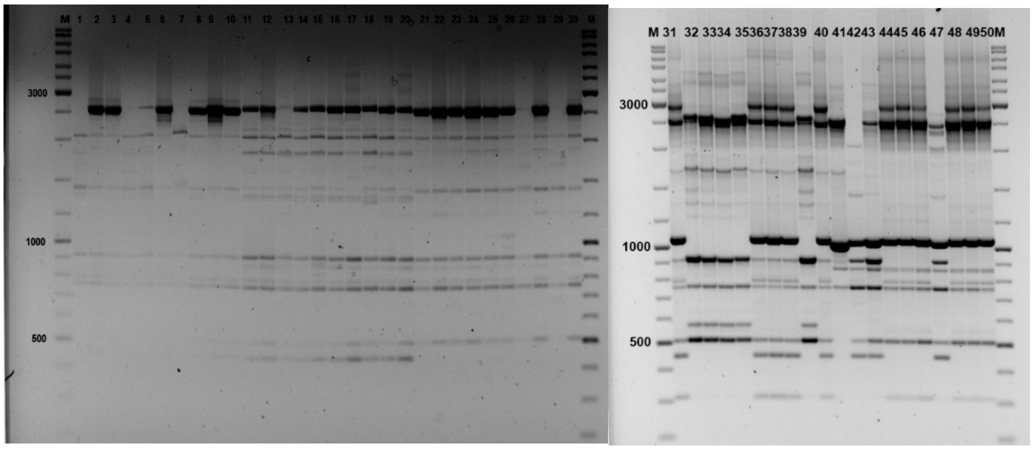

Figure S5 – inter Primer Binding Site (iPBS) profiling of individual DNA samples from *R. quadrifida* populations using primer 2228. M - Thermo Scientific GeneRuler DNA Ladder Mix (100–10,000 bp). Samples: 1-10 IVA-1 population, 11-20 IVA-2 population, 21-30 КОК population, 31-40 LIN-1 population, 41-50 LIN-2 population.

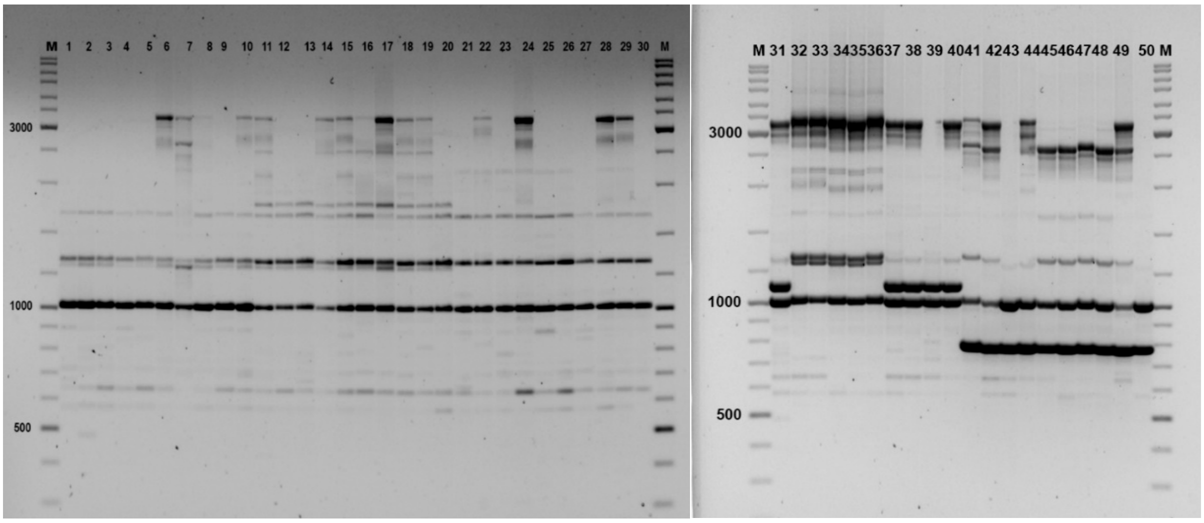

Figure S6 – inter Primer Binding Site (iPBS) profiling of individual DNA samples from *R. quadrifida* populations using primer 2229. M - Thermo Scientific GeneRuler DNA Ladder Mix (100–10,000 bp). Samples: 1-10 IVA-1 population, 11-20 IVA-2 population, 21-30 KOK population, 31-40 LIN-1 population, 41-50 LIN-2 population.

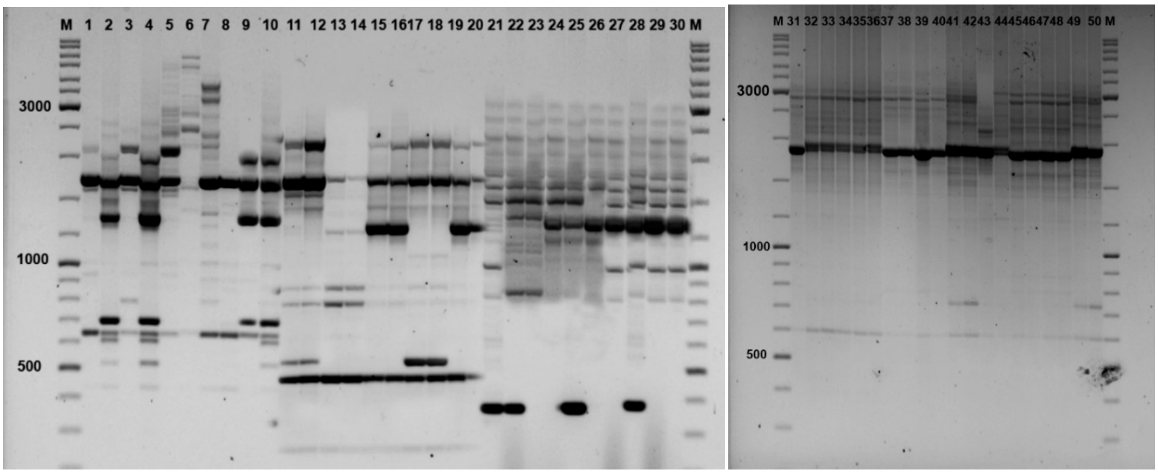

Figure S7 – inter Primer Binding Site (iPBS) profiling of individual DNA samples from *R. quadrifida* populations using primer 2230. M - Thermo Scientific GeneRuler DNA Ladder Mix (100–10,000 bp). Samples: 1-10 IVA-1 population, 11-20 IVA-2 population, 21-30 KOK population, 31-40 LIN-1 population, 41-50 LIN-2 population.

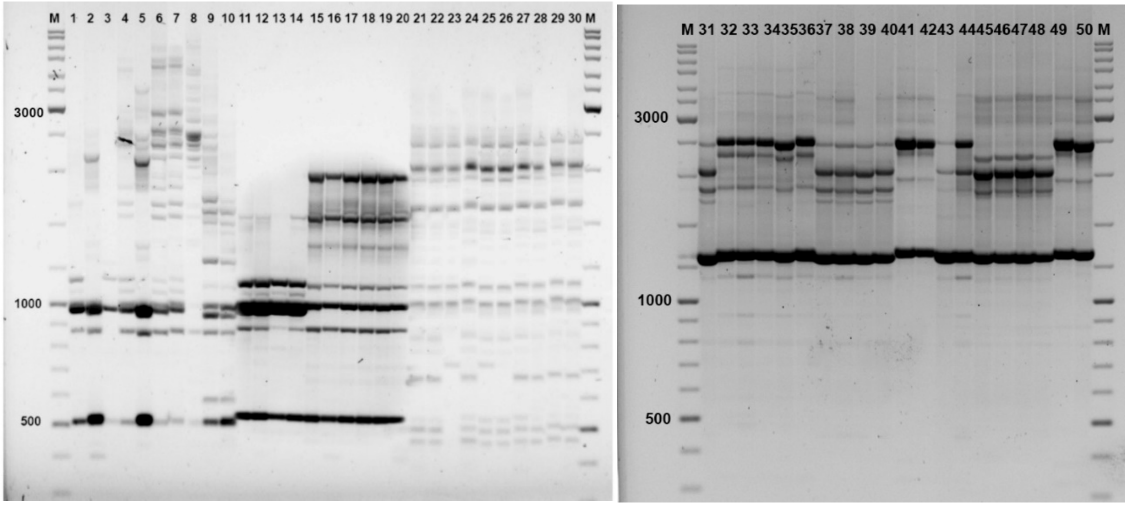

Figure S8 – inter Primer Binding Site (iPBS) profiling of individual DNA samples from *R. quadrifida* populations using primer 2232. M - Thermo Scientific GeneRuler DNA Ladder Mix (100–10,000 bp). Samples: 1-10 IVA-1 population, 11-20 IVA-2 population, 21-30 KOK population, 31-40 LIN-1 population, 41-50 LIN-2 population.

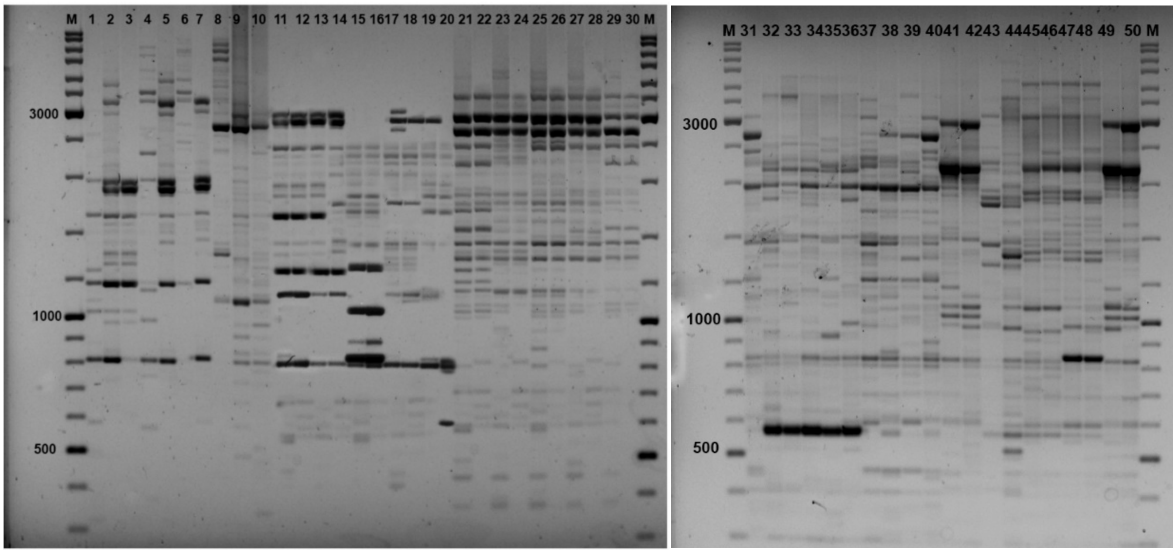

Figure S9 – inter Primer Binding Site (iPBS) profiling of individual DNA samples from *R. quadrifida* populations using primer 2240. M - Thermo Scientific GeneRuler DNA Ladder Mix (100–10,000 bp). Samples: 1-10 IVA-1 population, 11-20 IVA-2 population, 21-30 KOK population, 31-40 LIN-1 population, 41-50 LIN-2 population.

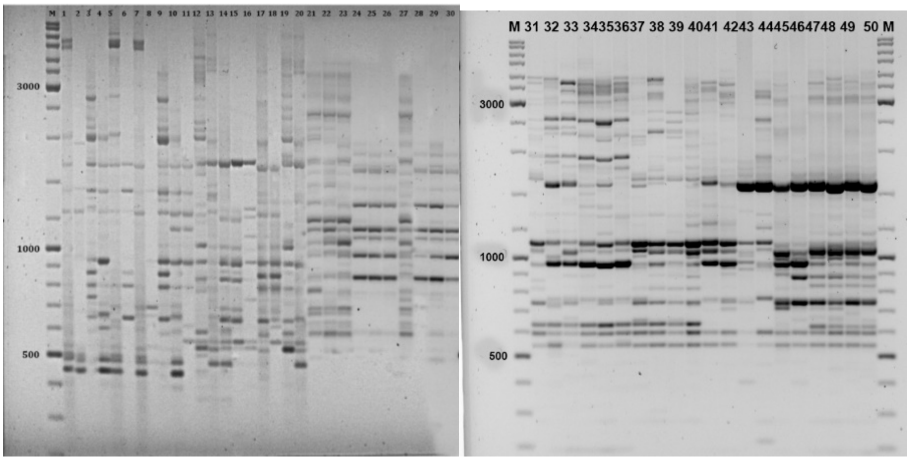

Figure S10 – inter Primer Binding Site (iPBS) profiling of individual DNA samples from *R. quadrifida* populations using primer 2241. M - Thermo Scientific GeneRuler DNA Ladder Mix (100–10,000 bp). Samples: 1-10 IVA-1 population, 11-20 IVA-2 population, 21-30 КОК population, 31-40 LIN-1 population, 41-50 LIN-2 population.

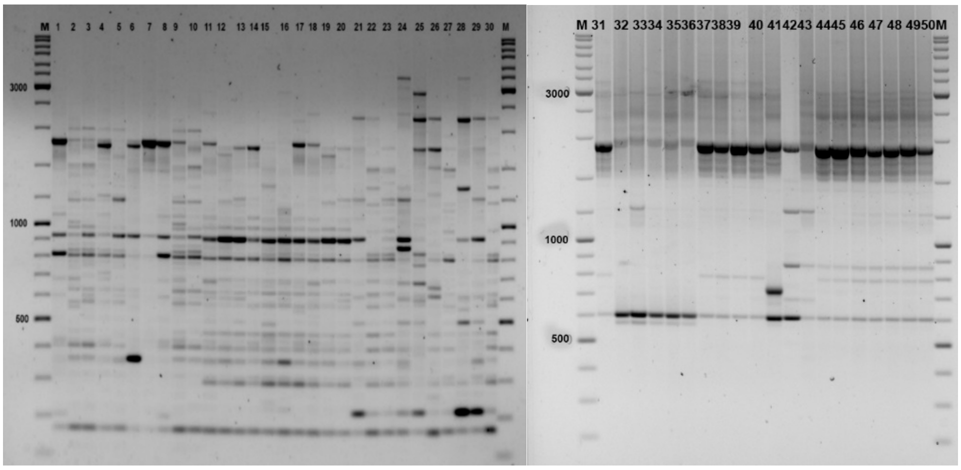

Figure S11 – inter Primer Binding Site (iPBS) profiling of individual DNA samples from *R. quadrifida* populations using primer 2300. M - Thermo Scientific GeneRuler DNA Ladder Mix (100–10,000 bp). Samples: 1-10 IVA-1 population, 11-20 IVA-2 population, 21-30 КОК population, 31-40 LIN-1 population, 41-50 LIN-2 population.

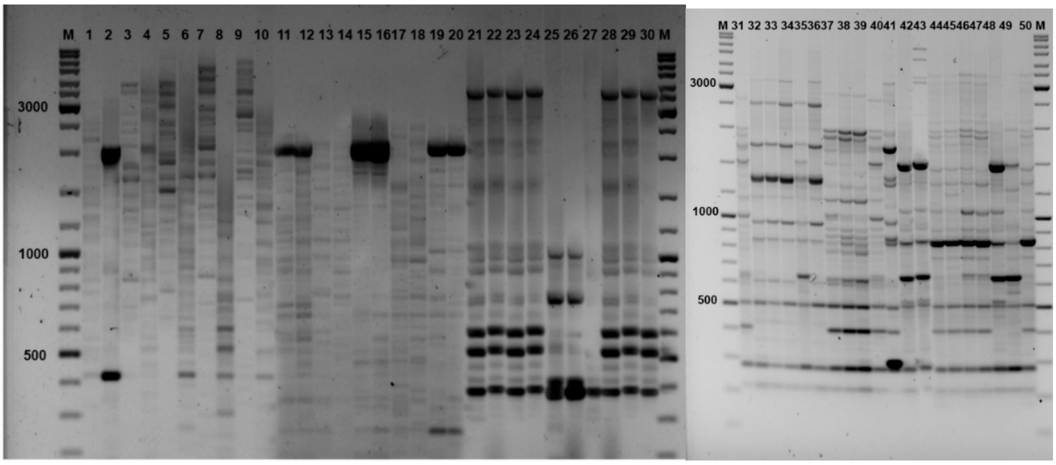

Figure S12– inter Primer Binding Site (iPBS) profiling of individual DNA samples from *R. quadrifida* populations using primer 2395. M - Thermo Scientific GeneRuler DNA Ladder Mix (100–10,000 bp). Samples: 1-10 IVA-1 population, 11-20 IVA-2 population, 21-30 КОК population, 31-40 LIN-1 population, 41-50 LIN-2 population.
